# Apolipoprotein D alleviates glucocorticoid-induced osteogenesis suppression in bone marrow mesenchymal stem cells via the PI3K/Akt pathway

**DOI:** 10.1186/s13018-020-01824-1

**Published:** 2020-08-08

**Authors:** Rong-Hua Yu, Xiang-yang Zhang, Wei Xu, Zhi-kun Li, Xiao-dong Zhu

**Affiliations:** Department of Orthopedics, Tongren Hospital, School of Medicine, Shanghai JiaoTong University, 1111 Xianxia Road Shanghai, Shanghai, 200336 P.R. China

**Keywords:** Apod, MSCs, PI3K/Akt, Osteogenesis

## Abstract

**Background:**

To clarify the role of apolipoprotein D (Apod) in alleviating glucocorticoid-induced osteogenesis suppression in bone marrow mesenchymal stem cells (MSCs) via the PI3K/Akt pathway, thus influencing the progression of osteoporosis (OP).

**Methods:**

Osteogenesis in MSCs was induced by dexamethasone (DEX) stimulation. Dynamic expressions of Apod in MSCs undergoing osteogenesis for different time points were determined by qRT-PCR. Relative levels of osteogenesis-associated genes, including ALP, RUNX2, and Osterix, in DEX-induced MSCs overexpressing Apod or not were examined. Moreover, the protein level of RUNX2, ALP, and Osterix; ALP activity; and mineralization ability influenced by Apod in osteogenic MSCs were assessed. At last, the potential influences of Apod on the PI3K/Akt pathway were identified through detecting the expression levels of PI3K and Akt in MSCs by Western blot.

**Results:**

Apod was time-dependently upregulated in MSCs undergoing osteogenesis. DEX induction downregulated ALP, RUNX2, and Osterix and attenuated ALP activity and mineralization ability in MSCs undergoing osteogenesis, which were partially reversed by overexpression of Apod. In addition, Apod overexpression upregulated the reduced levels of PI3K and Akt in DEX-induced MSCs.

**Conclusion:**

Apod alleviates glucocorticoid-induced osteogenesis suppression in MSCs via the PI3K/Akt pathway, thus protecting the progression of OP.

## Background

Glucocorticoid-induced osteoporosis (GIOP) is a common disease following long-term use or large doses of glucocorticoids [1, 2]. GIOP enhances susceptibility to bone fractures [3]. Attenuated bone formation and accelerated bone resorption lead to imbalanced progression, which is the major pathogenic reason for osteoporosis. Some drugs are reported to induce the occurrence of OP [4]. Among them, GIOP is prevalent. Even the administration of physiological doses of glucocorticoid (GC) can lead to bone loss. Menopausal women and men over 50 years of age are the risk population of OP. In particular, patients with joint diseases with long-term, continuous GC treatment are the highest-risk population of OP [4].

Apolipoprotein D (ApoD) is a 29-kDa glycoprotein highly conserved lipocalin known for its antioxidant and neuroprotective functions [5]. Unlike other apolipoproteins, they are essentially produced in the liver and intestine. Previous studies reported that ApoD was upregulated in many nervous system diseases (Alzheimer’s disease [6], Parkinson’s disease [7], and stroke [8]). Martineau et al. [9] reported that ApoD deficiency is associated with high bone turnover, low bone mass, and impaired osteoblastic function in aged female mice. Ishii et al. [10] found that ApoD gene expression was increased after osteogenic differentiation. Similar observations were reported in the murine MSC-like cell line C3H10 [11] and in mouse primary osteoblasts [12].

Studies have found that bone cells can participate in the regulation of biological activities of osteoblasts and osteoclasts through activating certain pathways [13]. Activation of the PI3K/Akt pathway in bone cells has been demonstrated to accelerate bone formation by stimulating osteogenesis and osteoblast resorption [14]. Meanwhile, it positively mediates osteogenesis in mesenchymal stem cells (MSCs) [15, 16]. PI3K/Akt was required for chondrocyte and osteoblast differentiation, and inhibition of PI3K/Akt could inhibit longitudinal bone growth [17].

This paper mainly analyzed the potential function and potential mechanism of Apod in affecting osteogenic differentiation of MSC osteogenesis.

## Materials and methods

### Cell culture and osteogenesis induction

MSCs were purchased from Fuyuan Bio (Shanghai, China) that were isolated from two donors and cultured in low-glucose DMEM (Gibco) supplemented with 10% FBS (Gibco) and 1% penicillin/streptomycin and cultured in a humidified incubator at 37 °C and 5% CO_2_. Cell passage was conducted every 2 days. For osteogenesis induction, MSCs were inoculated in a 6-well plate at a density of 2.0 × 10^5^/mL. Osteogenic induction was applied as follows: DMEM + 10% FBS + 1% penicillin-streptomycin + 10^−7^ mol/L DEX + 10 mmol/L β-glycerophosphate + 50 μg/mL ascorbic acid. Osteogenesis was induced for 7–14 days. The medium was replaced every 3 days.

### DEX treatment

Cell suspension was prepared at a density of 5 × 10^4^/mL. A total of 100 μL (containing 5 × 10^3^ cells) of suspension was applied in each well of a 96-well plate. After overnight cell adherence, medium containing 1 μM DEX was replaced.

### Cell transfection

Cell transfection was conducted using Lipofectamine 2000 (Invitrogen, Carlsbad, CA, USA). Transfection vectors were provided by GenePharma (Shanghai, China). MSCs were infected with the Apod-expressing lentivirus or control lentivirus in the presence of 8 μg/mL polybrene, and the resulting cell lines were named MSC-Apod and MSC-GFP, respectively.

### Quantitative reverse transcriptase polymerase chain reaction assay (q-RTPCR) analysis

Total RNA was extracted using TRIzol reagent (Invitrogen, Carlsbad, CA, USA). Total RNA was quantified by measuring the absorbance at 260 nm (NanoDrop 2000; Thermo Fisher Scientific, MA, USA). Single-strand cDNA was synthesized by reverse transcription with a Superscript II Reverse Transcriptase Kit and oligo dT primers (Invitrogen, Carlsbad, CA, USA) and amplified by reverse transcription PCR with iQ SYBR Green Supermix (Biorad, Hercules, CA, USA). qPCR was performed using SYBR Green PCR Master Mix (Applied Biosystems), following the manufacturer’s instructions. The expression levels of the target genes were calculated relative to housekeeping GAPDH using Stratagene Mx3000P software (Applied Biosystems) with the 2^−ΔΔCt^ equation. The primer sequences were listed in Table 1.

**Table 1:** Sequences of primers for quantitative real-time PCR

Gene	Forward primer (5′-3′)	Reverse primer (3′-5′)
GAPDH	TTCTTTTGCGTCGCCAGCCGA	GTCACCACCCGCCCAATACGA
RUNX2	GGGTAAGACTGGT-CATAGGACC	CCCAGT-ATGAGAGTAGGTGTCC
ALP	ACCACCACGAGAGTGAACCA	CGTTGTCTGAGTACCAGTCCC
Osterix	AGGAGGCACAAAGAAGCCATAC	AGGGAAGGGTGGGTAGTCATT
Apod	TTAACCTCACAGAGCCTGCC-3	GAGTCCACTGTTTCTGGAGGG

### Western blot

The total proteins were extracted by radioimmunoprecipitation assay (RIPA) (Solarbio, Beijing, China) lysate and then separated by sodium dodecyl sulfate-polyacrylamide gel electrophoresis (SDS-PAGE). The separated proteins were transferred onto the polyvinylidene difluoride membranes. The membranes were blocked with 5% nonfat milk dissolved in Tris-buffered saline with 0.05% Tween-20 (TBS-T) for 1 h at room temperature. The membranes were then incubated overnight at 4 °C with primary antibodies against Apod (1:2000), Osterix (1:1000), ALP (1:4000), RUNX2 (1:2000), p-PI3K (1:2000), PI3K(1:1000), p-Akt (S437, 1:2000), Akt (1:2000), and GAPDH (1:8000). Finally, the membranes were incubated for 1 h with horseradish peroxidase (HRP)-conjugated secondary antibodies (1:500) and visualized using an enhanced chemiluminescence system, according to the manufacturer’s instructions.

### Alkaline phosphatase (ALP) activity determination

MSCs were washed with pre-cold PBS for three times and lysed in pre-cold 1% Triton X-100 on ice for 30 min. The cell lysate was subjected to ALP activity determination, and the value at 405 nm was normalized to that of total protein concentration.

### ALP staining

MSCs were washed with PBS twice and reacted in 70% ethanol for 10 min and ALP buffer (0.15 M NaCl, 0.15 M Tris-HCl, 1 mM MgCl_2_, pH 9.5) for 15 min. Subsequently, cells were cultured in the NBT-BCIP solution at 37 °C, in the dark for 30 min. Images were captured under a microscope.

### Alizarin red staining (ARS)

MSCs were induced for 14-day osteogenesis. Cells were washed, fixed in 95% ethanol for 14 min, and dyed in 0.1% ARS-Tris-HCL solution (pH 4.3). Visible mineralized nodules were captured under an inverted microscope.

### Bioinformatic analysis

To reveal the potential mechanism of Apod, the Search Tool for the Retrieval of Interacting Genes (STRING, http://string-db.org) database was searched the adjacent molecular of Apod. The Molecular Complex Detection (MCODE) app was used to analyze protein-protein interaction (PPI) networks [18]. The PPI networks were mapped using the Cytoscape software (version 3.7.2, http://www.cytoscape.org/). The cutoff criteria for the module genes were set as follows: degree cutoff ≥ 2, node score cutoff ≥ 2, K-core ≥ 2, and max depth = 100. Subsequently, Gene Ontology (GO) and KEGG pathway enrichment analysis of target genes were conducted via the Database of Annotation, Visualization, and Integration Discovery (DAVID; version 6.8; http://david.abcc.ncifcrf.gov/). GO terms include biological process (BP), cellular component (CC), and molecular function (MF).

### Statistical analyses

Data were expressed as mean ± standard deviation (SD). SPSS 20.0 software (IBM, Armonk, NY, USA) was applied for data analysis. The *t* test and one-way ANOVA (followed by Tukey’s post hoc test) was used for analyzing the differences between two groups and multiple groups, respectively. *P* < 0.05 indicated a significant difference.

## Results

### Apod level was upregulated during osteogenesis

**Fig. 1:**
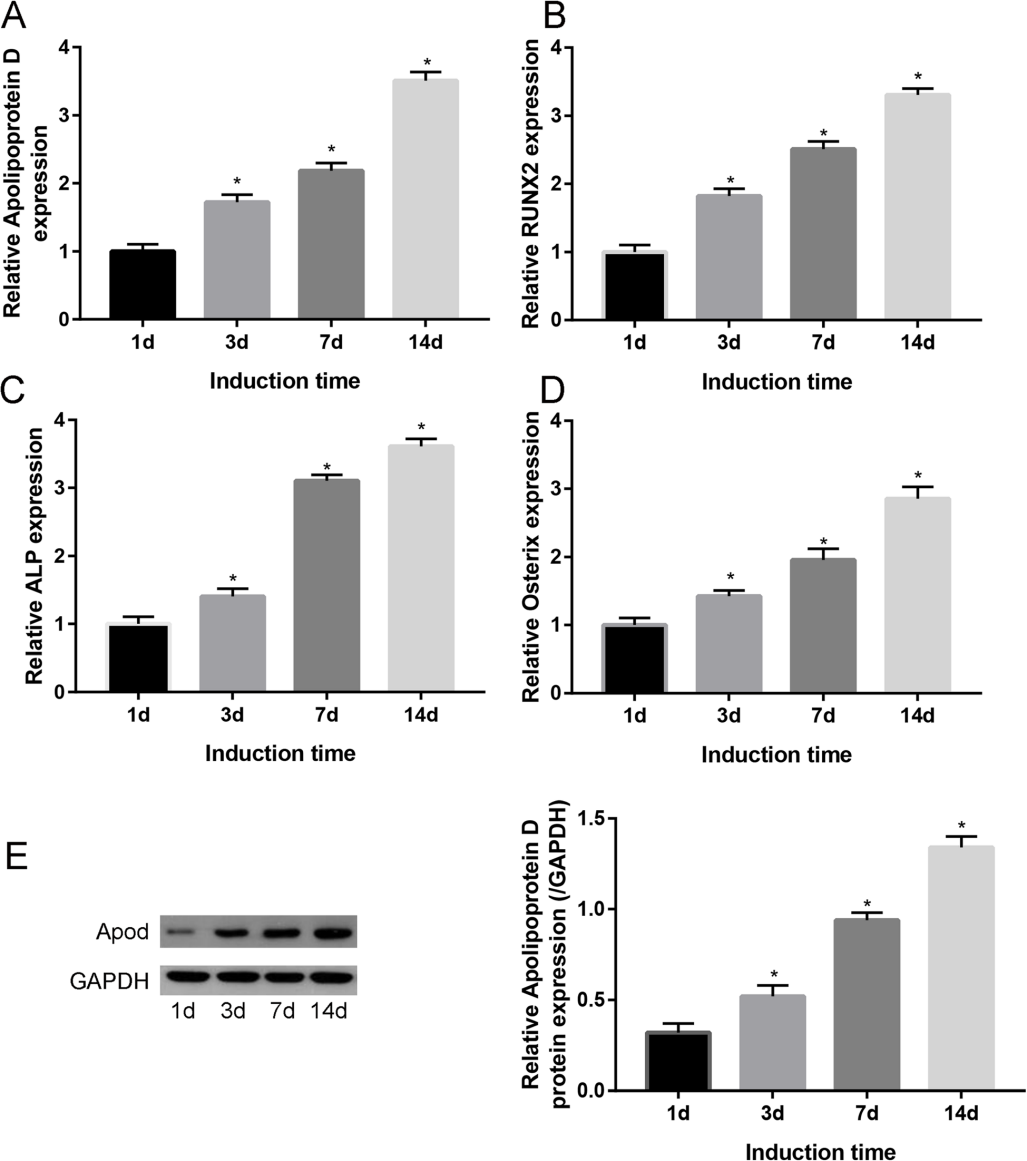
Dynamic change of Apod (**a**), RUNX2 (**b**), ALP (**c**), and Osterix (**d**) in MSC osteogenic differentiation for 1, 3, 7, and 14 days. **e** Western blot analyses of Apod in MSC osteogenic differentiation for 1, 3, 7, and 14 days

Compared with osteogenesis induced in MSCs for 1 day, after osteogenesis induced in MSCs for 3, 7, and 14 days, Apod (Fig. 1a), RUNX2 (Fig. 1b), ALP (Fig. 1c), and Osterix (Fig. 1d) was time-dependently upregulated. Those results indicated that Apod was involved in the osteogenic differentiation of MSCs.

What is more, Western blot was performed and revealed that after osteogenesis is induced in MSCs for 1, 3, 7, and 14 days, Apod was time-dependently upregulated (Fig. 1e), suggesting the involvement of Apod in osteogenesis.

### Bioinformatic analysis

**Fig. 2:**
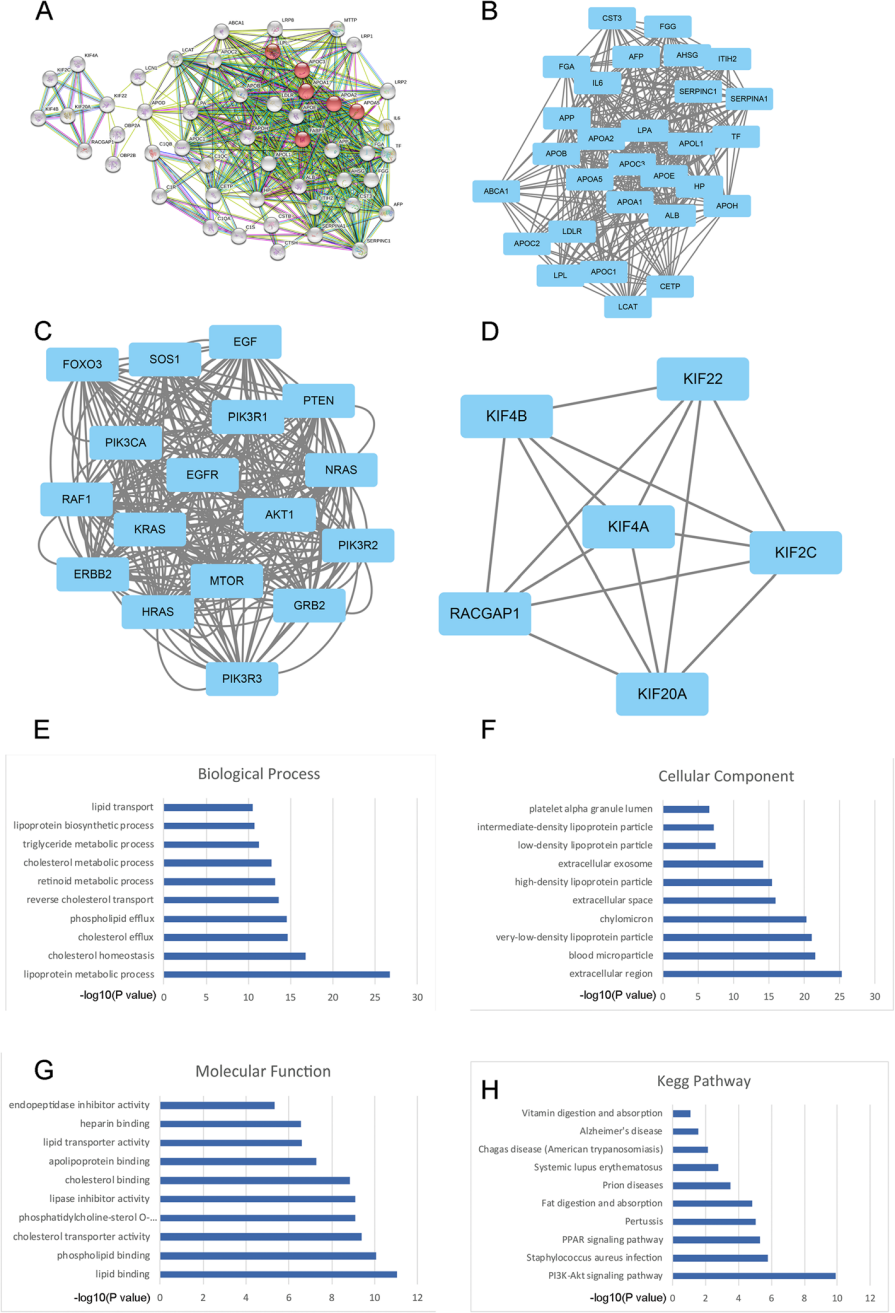
Protein-protein interaction of Apod and targeting genes (**a**). MCODE models 1 (**b**), 2 (**c**), and 3 (**d**) of the protein-protein interaction. Biological process (**e**), cellular component (**f**), molecular function (**g**), and the KEGG pathway (**h**) of the Apod target genes

PPI was performed and found that Apod is the center of the total PPI. Then, we used MCODE to screen the modules of the gene interaction network, and three modules were shown in Fig. 2a–d. The score of the top 1 module includes FGA, APP, AFP, APOA5, and APOA2. The score of the top 2 module mainly includes PI3CA, EGFR, Akt1, PI3KR2, and PI3KR3. The score of the top 3 module includes KIF4B, KIF4A, KIF22, KIF2C, KIF20A, and RACGAP1.

**Fig. 3:**
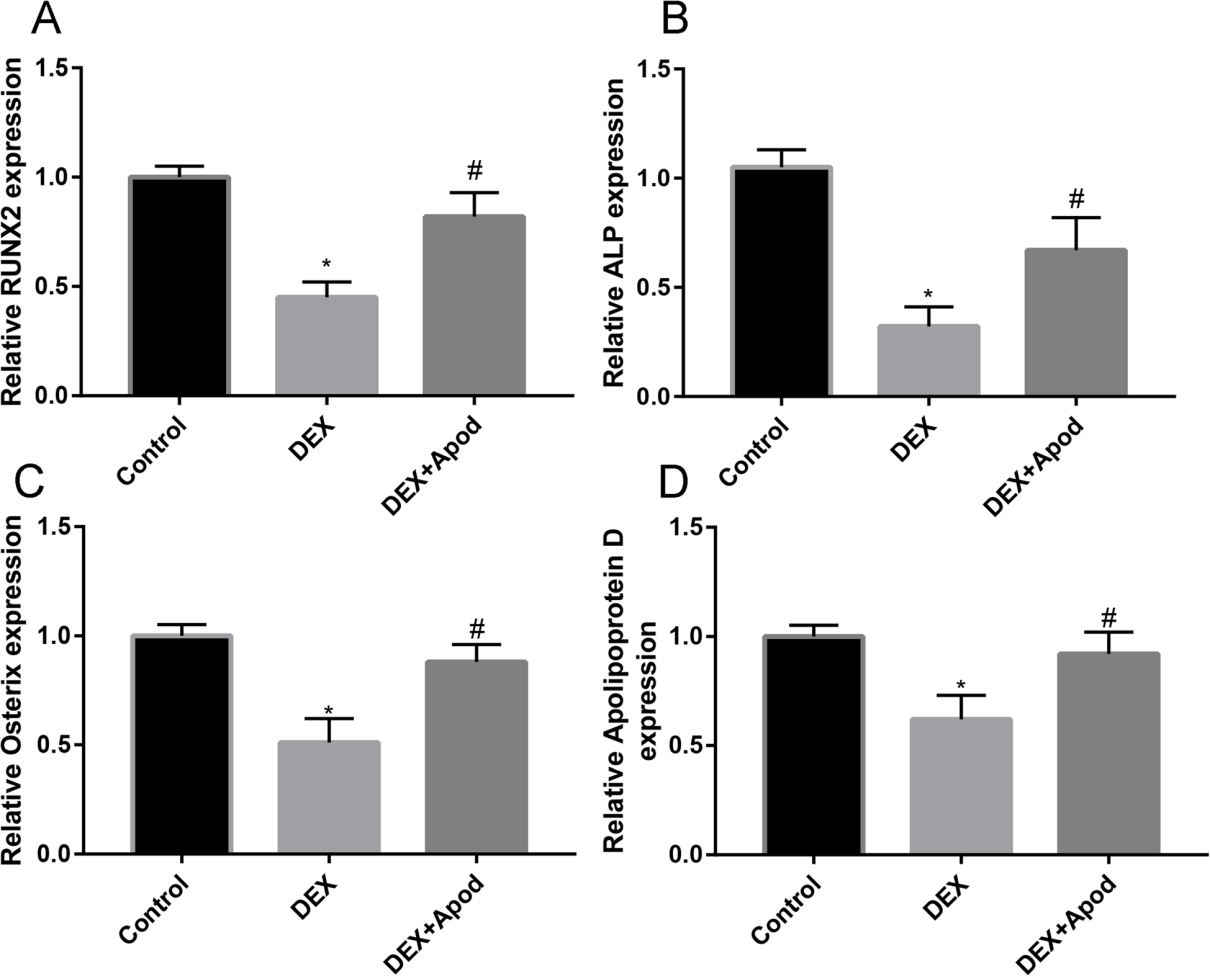
Overexpression of Apod reversed the downregulated RUNX2 (**a**), ALP (**b**), Osterix (**c**), and Apod (**d**)

The functional annotation of the target genes was clarified using the DAVID 6.8 online tool. Biological process analysis indicated that these target genes were significantly enriched in lipoprotein metabolic process, cholesterol homeostasis, and cholesterol efflux (Fig. 2d). For cellular components, the target genes were enriched in the extracellular region, blood microparticle, and very low-density lipoprotein particle (Fig. 3e). For molecular function, target genes were significantly enriched in lipid binding, phospholipid binding, and cholesterol transporter activity (Fig. 3f). For the KEGG pathway, target genes mainly enriched in the PI3K-Akt signaling pathway (Fig. 3g).

### Overexpression of Apod reversed the downregulated osteogenesis-associated genes in DEX-induced MSCs

To elucidate the potential function of Apod in MSC osteogenesis, cells were induced in medium containing 1 μM DEX. DEX induction markedly downregulated relative levels of RUNX2 (Fig. 3a), ALP (Fig. 3b), and Osterix (Fig. 3c), which were partially reversed by the overexpression of Apod. Moreover, we found that DEX induction markedly downregulated relative levels of Apod, and overexpression of Apod significantly upregulated the expression of Apod (Fig. 3d), which indicated that the cell transfection was successful.

### Overexpression of Apod alleviated DEX-induced osteogenesis suppression in MSCs

**Fig. 4:**
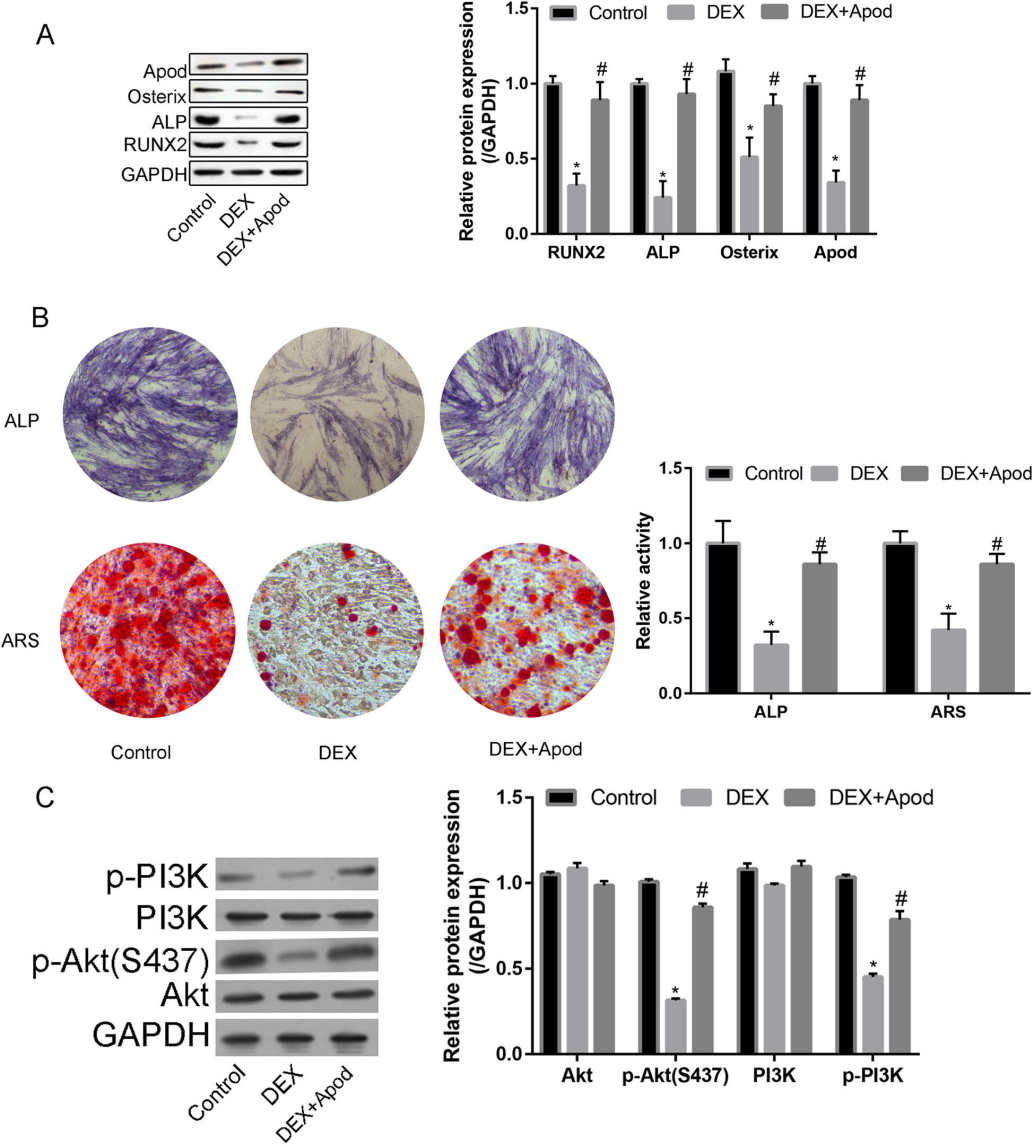
Overexpression of Apod alleviated DEX-induced osteogenesis suppression in MSCs. MSCs were treated with control, DEX, and DEX+Apod overexpression. **a** Protein level of RUNX2, ALP, Osterix, and Apod. **b** ALP activity and ARS staining. **c** Apod-regulated MSC osteogenesis via the PI3K/Akt signaling pathway. Protein level of PI3K, p-PI3K, Akt, and p-Akt in MSCs treated with blank control, DEX, and DEX+Apod overexpression

The downregulated protein level of RUNX2, ALP, Osterix, and Apod after DEX treatment was reversed by the overexpression of Apod in MSCs, which was similar to its changing trend at the mRNA level (Fig. 4a).

In addition, ALP and ARS activity decreased following DEX treatment, and it was further elevated by overexpression of Apod (Fig. 4b).

### Apod regulated MSC osteogenesis via the Wnt pathway

After DEX treatment, protein levels of p-PI3K and p-Akt (S437) were downregulated. However, their downregulation was reversed by the overexpression of Apod (Fig. 4c). It is believed that Apod alleviated GC-induced osteogenesis suppression in MSCs.

## Discussion

GC is one of the most commonly applied drugs. However, the long-term application of GC would lead to many complications, including OP, osteonecrosis, and metabolic syndrome [19, 20]. GIOP is the most common subtype of OP and is a metabolic skeletal disorder [21]. However, the mechanism of GIOP was not fully understood.

In our analysis, Apod was upregulated during osteogenesis and accelerated osteogenesis in MSCs. DEX significantly reduced ALP and ARS activity; these effects were reversed by extra adding Apod. Those results found that Apod could regulate the osteogenic differentiation of MSCs. Martineau et al. [9] revealed that when Apod was overexpressed, the osteogenic differentiation ability was increased. Oxidative damage plays a vital role in inducing GIOP. Previous studies found that superoxide dismutase (SOD) and catalase were increased during osteogenic differentiation of hBMSC. ApoD was increased in response to oxidative stress in the brain and has been suggested to function as an antioxidant in the brain [22].

Apod emerges as an evolutionarily conserved anti-stress protein that is induced by oxidative stress and inflammation and may prove to be an effective therapeutic agent against a variety of neuropathologies, and even against aging [23]. What is more, oxidative stress induces Apod overexpression in the hippocampus during aging and Alzheimer’s disease [24].

Moreover, we used bioinformatic analysis to reveal the potential mechanism of Apod in regulating the osteogenic differentiation of MSCs. We found that target genes of Apod mainly enriched in lipoprotein metabolic process, and the KEGG pathway revealed that the PI3K/Akt signaling pathway was the most enriched pathway. DEX could significantly reduce the p-PI3K and p-Akt expression, while Apod could reverse the downregulation of p-PI3K and p-Akt. Taken together, Apod could reverse the osteogenic inhibitory effect of DEX on osteogenic differentiation of MSCs through the PI3K/Akt signaling pathway.

Our findings uncovered that downregulated osteogenesis-associated genes in DEX-induced MSCs were partially reversed by the overexpression of Apod, as well as the attenuated ALP activity and mineralization ability. Notably, Apod overexpression activated the PI3K/Akt pathway in DEX-induced MSCs.

## Conclusions

Apod alleviates glucocorticoid-induced osteogenesis suppression in MSCs *via* the PI3K/Akt pathway, thus protecting the progression of GIOP.

## Data Availability

The datasets used and/or analyzed during the current study are available from the corresponding authors upon reasonable request.

## Abbreviations

Apod: Apolipoprotein D
MSCs: Mesenchymal stem cells
OP: Osteoporosis
DEX: Dexamethasone
GIOP: Glucocorticoid-induced osteoporosis
GC: Glucocorticoid
DMEM: Dulbecco’s modified Eagle’s medium
FBS: Fetal bovine serum
q-RTPCR: Quantitative reverse transcriptase-polymerase chain reaction assay
SDS-PAGE: Sodium dodecyl sulfate-polyacrylamide gel electrophoresis
HRP: Horseradish peroxidase
ALP: Alkaline phosphatase
ARS: Alizarin red staining
STRING: Search Tool for the Retrieval of Interacting Genes
MCODE: Molecular Complex Detection
PPI: Protein-protein interaction
DAVID: Database of Annotation, Visualization, and Integration Discovery
BP: Biological process
CC: Cellular component
MF: Molecular function
SPSS: Statistical Product and Service Solutions
SOD: Superoxide dismutase
